# Fabrication and Optical Properties of Water Soluble CdSeS Nanocrystals Using Glycerin as Stabilizing Agent

**DOI:** 10.1371/journal.pone.0077253

**Published:** 2013-10-21

**Authors:** Fengrui Jiang, Guolong Tan

**Affiliations:** State Key Laboratory of Advanced Technology for Materials Synthesis and Processing, Wuhan University of Technology, Wuhan, China; Texas A&M University, United States of America

## Abstract

Herein we present an unusual phosphine-free method to fabricate water soluble CdSeS nanocrystals in cubic structure. In this method, glycerin was used as a stabilizing agent replacing tri-n-octylphosphine oxide (TOPO). Water solution of Na_2_SeO_3_ in polyethylene glycol was utilized as Se source. 3-Mercaptopropionic acid (MPA) provides S source. The phosphine-free Se and S sources were found to be highly reactive and suitable for the synthesis of CdSeS nanocrystals. XRD and HRTEM images confirm the formation of CdSeS nanocrystals in zinc blende structure. The absorption peaks on UV-vis spectra of as-prepared CdSeS nanocrystals are tunable from 330 nm to 440 nm, which blue shifts to shorter wavelength side in comparison with that of pure CdSe nanocrystals. The cubic CdSeS nanocrystals demonstrate narrow PL emissions spectra between 464 and 615 nm. Transmission electron microscopy images show the uniformity for the size distribution of the ternary QDs. Series water soluble CdSe_1–x_S_x_ (x = 0∼1) nanocrystals have also been synthesized using Na_2_SeO_3_ and Na_2_S solution as the Se-S co-sources. Tunable band gap energies of CdSe_1–x_S_x_ (x = 0∼1) nanocrystals upon chemical composition x have been achieved, the gap ranges from 290 nm to 558 nm.

## Introduction

Over the past decades, scientists have discovered new species ranging between molecules and solids with unique size dependent physical and chemical properties [1]–[3]. One prominent example are colloidal semiconductor nanocrystals (NCs) [4], which have found their way into various applications such as LEDs, [5]–[8] solar cells [9], [10] and fluorescent labeling [11], [12]. To achieve the widespread use of QD-based devices and systems, it would be important to develop safe, economical, and environmentally friendly large-scale syntheses of high-quality QDs.

For the synthesis of colloidal II-VI QDs, earlier efforts focused more on binary systems, such as CdSe and CdS QDs, and recent efforts more on ternary systems, such as CdTeSe [13]–[15], CdSeS [16]–[18], ZnCdSe, and ZnCdS QD alloys [19]–[25]. For the ternary QDs, bandgap engineering can be achieved via control of their sizes, constituent stoichiometry, and internal structures (such as homogeneous vs. gradient). For homogeneous ternary QDs, their compositions play an essential role affecting both the confinement potential and the interfacial strain; thus, their band gap energy can be tuned, even at a constant size [26]. For example, alloyed CdSeS nanocrystals can be tuned readily to emit in the wavelength range of 480–540 nm, which is not easily achieved with binary CdSe or CdS QDs alone [4], [27]–[30]. The ternary QDs reported by other research groups were from hot-injection approaches using of Tri-n-octylphosphine (TOP)/tri-n-octylphosphine oxide (TOPO) or other long chain organic compounds [26], [31] as the capping agent at high temperature.

The high-temperature synthesis method usually involves the reaction of a cadmium compound with a phosphine complex of selenium and sulfur [4], [30]–[33]. The product often exhibits high PL QY and photostability. However, the organic compounds and phosphines (e.g., trioctylphosphine (TOP) and tributylphosphine (TBP)) are usually toxic and expensive. The Se and S precursor is still almost invariably a Se- or S-phosphine complex. The use of such capping agents requires very stringent experimental conditions, such as an inert atmosphere and high temperature [15]. Meanwhile, these phosphines or other organic compounds are not water soluble. Therefore, it is still a challenge to develop new synthesis routes leading to water soluble ternary semiconductor nanocrystals with uniform size by a phosphine-free method, which is affordable and could be easily scaled up. One of the most important aspects is the capping agent, which could stabilize nanocrystals in solutions. In contrast, by using polymeric stabilizers to synthesize nanocrystals is an easy, safe way and is much cheaper than that employing TOP/TOPO as the capping agent. In general, polymer stabilizer only requires ambient laboratory conditions [34], [35].

In this work, glycerin has been used to replace TOPO as a new kind of capping agent and phosphine-free Na_2_SeO_3_ solution as Se source, 3-mercaptopropionic acid (MPA) and Na_2_S as the S source to produce CdSeS nanocrystals, which is water soluble and could be stabilized in the solution for several months without having its color and optical properties changed.

## Results and Discussion

### 1.1. Structure determination

**Figure 1:**
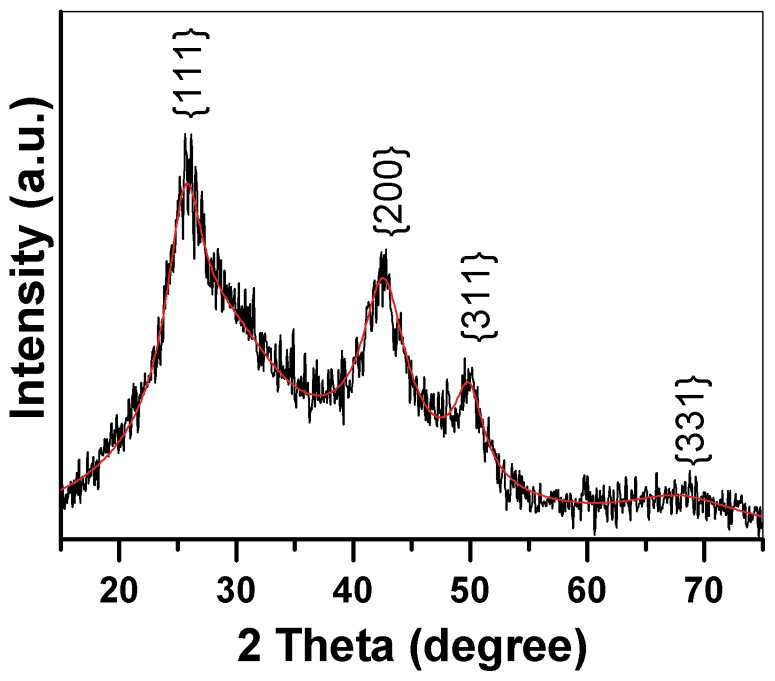
XRD pattern of as-prepared CdSe_0.2_S_0.8_ nanocrystals.

CdSeS nanocrystals were thus produced through the reaction of Cd source with Se source from Na_2_SeO_3_ and S source from MPA. Figure 1 shows the X-ray diffraction pattern of CdSeS nanocrystals. It can be seen that the as-prepared CdSeS nanocrystals exhibit cubic structure. All the diffraction peaks locate within the angle positions between that of cubic CdSe (JCPDS 65–2891) and cubic CdS (JCPDS 65–2887). The diffraction peaks at 25.47°, 42.22° and 49.91° correspond to the lattice planes of (111), (220) and (311), suggesting that the nanoparticles are in zinc blende structure and are in good agreement with the reported data on CdSe_1–x_S_x_ nanoparticles [17], [36]–[39]. The lattice parameter of the CdSeS nanocrystals has been determined to be a = 1.9769Å from the XRD pattern through Bragg equation. By using the formula of (a_1_ (CdS)  = 1.9266Å, a_2_ (CdSe)  = 2.0144Å; a_x_ is the lattice parameter of the as-prepared CdSe_1–x_S_x_) [17], the chemical composition value of x is calculated to be 0.8. Thus the chemical formula of CdSe_1–x_S_x_ can be now expressed as CdSe_0.2_S_0.8_. Obviously, the diffraction peaks for *CdSe_0.2_S_0.8_* nanocrystals have been broaden, indicating the formation of ultrafine nanocrystals. The average particle size calculated from Scherer's formula was 4.5 nm. In fact, wurtzite and zinc blende structure have similar lattice structures, both of them own the same tetragonally positioned first nearest neighbors and nearly identical secondary nearest neighbors [40]. Therefore, XRD pattern is hard to discriminate zinc blende structure from wurtzite structure in the as-prepared CdSeS nanocrystals. The existence of the wurtzite structure could not be excluded in this broadened XRD pattern. However, the XRD pattern exhibits standard shape of zinc blende structure, which was considered to be the final phase of the as-prepared *CdSe_0.2_S_0.8_* nanocrystals and the evidence for the calculation of their lattice parameter. High resolution transmission electron microscopy (HRTEM) images of these CdSeS nanocrystals in the following section will prove that this hypothesis is correct.

### 1.2. Morphology and microstructure

**Figure 2:**
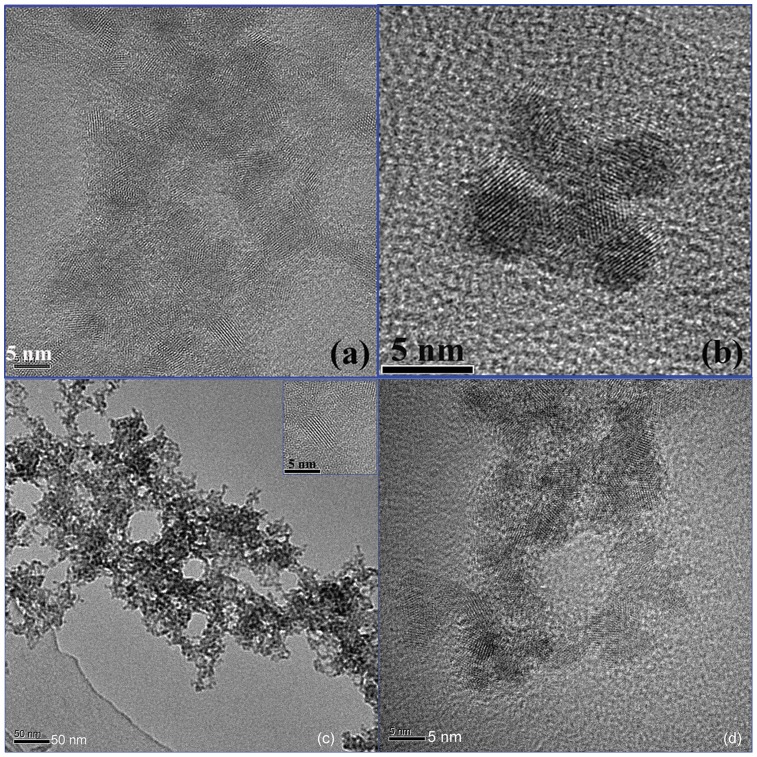
TEM and HRTEM images of as-prepared CdSe_0.2_S_0.8_ nanocrystals.

The microstructure and morphology of the as-prepared *CdSe_0.2_S_0.8_* nanocrystals are exhibited in Figure 2 (c) shows a low magnification TEM image of the *CdSe_0.2_S_0.8_* nanocrystals. It can be seen from Figure 2 (c) that glycerin forms a network frame, which hosts the *CdSe_0.2_S_0.8_* nanocrystals on branches. Some aggregation of the *CdSe_0.2_S_0.8_* nanocrystals on the network frame can be observed in Figure 2 (c) due to the high viscosity of the glycerin.

Figure 2 (a) (b) and (d) show the HRTEM images of as-prepared *CdSe_0.2_S_0.8_* nanocrystals using glycerin as the stabilizing agent. They clearly confirm the well order crystal structure of *CdSe_0.2_S_0.8_* nanocrystals. The lattice fringes are clearly seen in these nanocrystals, which are assigned to {111} and {200} lattice planes in zinc blende structure. The HRTEM images of individual nanocrystals further confirm the cubic structure feature of the as-prepared *CdSe_0.2_S_0.8_* nanocrystals using the glycerin as the stabilizing agent, being in good agreement with the XRD results. Lattice disorder and stacking faults are clearly seen in these particles, as being shown in Figure 2 (a) & (b). The particles are homogeneously distributed, ranging from 3 nm to 6 nm. The polymer with higher viscosity would induce big stress upon the surface of the nanocrystals, which could easily cause the formation of the defects and twin structures in the CdSeS nanocrystals. However, individual *CdSe_0.2_S_0.8_* nanocrystals could be still discriminated each other on these HRTEM images. Some particles are well separated; exhibiting monodispersive size distribution, as being shown in Figure 2.

### 1.3. UV-vis spectra

**Figure 3:**
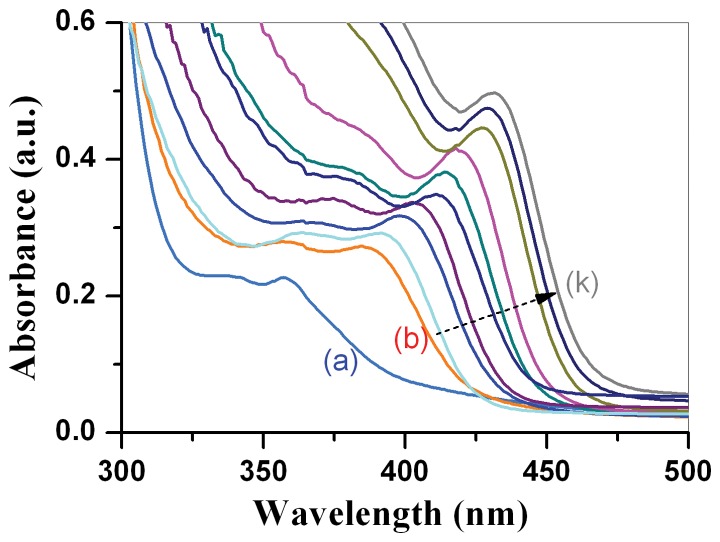
Tunable UV-Vis absorption spectra of CdSe_0.2_S_0.8_ nanocrysals upon particle size, the solution containing CdSe_0.2_S_0.8_ nanocrystals was heat treated at (a) 140°C for 5 min, (b)180°C for 5 min, (b)220°C for 5 min, … (j) 260°C for 4 hs; (k)260°C for 5 h.

Figure 3 shows the tunable UV – Vis absorption spectra of *CdSe_0.2_S_0.8_* nanocrystals upon different particle size, which was controlled by temperature and period of heat treatment. With control of the particle size of the *CdSe_0.2_S_0.8_* nanocrystals, the color of the *CdSe_0.2_S_0.8_* QDs could be tuned. The absorption spectrum showed a slight broadening caused by the size increase during the growth of the *CdSe_0.2_S_0.8_* nanocrystals, as being shown in Figure 3. The particle size increases with the temperature and period of heat treatment of the solution, inducing the absorption peaks red shift to longer wavelength side, as being shown in Figure 3.

There are two absorption peaks locating at 335 nm and 358 nm respectively for the *CdSe_0.2_S_0.8_* nanocrystals which were heat treated at 140°C for 5 minutes (Figure 3 (a)). The two peaks red shift to 356 nm and 388 nm for the *CdSe_0.2_S_0.8_* nanocrystals which were heat treated at 180°C for 5 minutes (Figure 3 (b)). These absorption peaks keep shifting to the longer wavelength side when *CdSe_0.2_S_0.8_* nanocrystals were heat treated at higher temperature or for longer period; until it reaches at 432 nm after *CdSe_0.2_S_0.8_* nanocrystals were heat treated at 260°C for 5 hours (Figure 3 (k)). This kind of red shift was caused by well-known quantum confinement effect. The higher is the heat treatment temperature, the larger is the particle size and thus smaller the band gap energies of the *CdSe_0.2_S_0.8_* nanocrystals, which leads to the red shift of the absorption peaks of the quantum dots.

XRD results and HRTEM images demonstrate that the as-prepared *CdSe_0.2_S_0.8_* nanocrystals were in zinc blende structure when glycerin was used as the stabilizing precursor. The band gap energy of CdSe is 1.714 eV [41], while that of cubic CdS is 2.50 eV. The band gap energy of bulk *CdSe_0.2_S_0.8_* should be located somewhere between 1.714 to 2.50 eV. However, when size of CdSe_0.2_S_0.8_ nanocrystals is smaller than its Bohr radius, the band gap will be enlarged due to quantum confinement effect. Therefore the UV visible absorption peaks didn't move to the conventional wavelength region within 500∼650 nm for pure CdSe nanocrystals due to the enlargement of its band gap by finer particle size. Instead the absorption peaks of our *CdSe_0.2_S_0.8_* nanocrystals are located within the wavelength range of 335 nm∼432 nm, which shouldn't reflect ultra-small particle size of CdSe nanocrystals, but actually is corresponding to the tunable band gap energies of *CdSe_0.2_S_0.8_* nanocrystals being caused by the chemical composition. These kinds of *CdSe_0.2_S_0.8_* being produced using glycerin as the stabilizing agent are water soluble.

**Figure 4:**
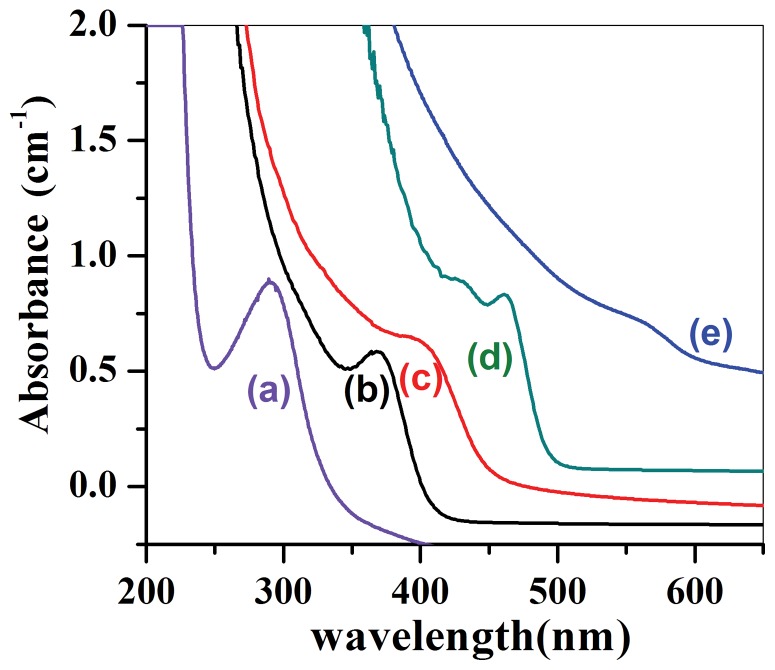
The UV absorption of CdSe_1–x_S_x_ nanocrystals in different Se/S ratio, (a) x = 1, (b) x = 0.75, (c) x = 0.5, (d) x = 0.25 and (e) x = 0.

Series CdSe_1–x_S_x_ nanocrystals were intentionally fabricated using Na_2_SeO_3_ and Na_2_S solution as the Se-S sources. The experimental process was the same as the above *CdSe_0.2_S_0.8_* nanocrystals except that MPA was replaced by Na_2_S to provide S source. The atomic ratio of Na_2_SeO_3_: Na_2_S was set to be 0∶1(x = 1), 0.25∶0.75(x = 0.75), 0.5∶0.5(x = 0.5), 0.75∶0.25 (x = 0.25) and 1∶0 (x = 0). Se-S mixed precursor was dropped into Cd precursor very slowly at a temperature of 260°C. All the specimens were heat treated at this temperature for 5 minutes. The UV visible absorption spectra of the as-prepared CdSe_1–x_S_x_ nanocrystals are demonstrated in Figure 4, where it can be seen that the absorption peaks red shift to longer wavelength side with the concentration of Se. There is an absorption peak locating at 290 nm for CdS (x = 1) nanocrystals, this peak red shifts to 368 nm and 401 nm for the composition of x = 0.75 and x = 0.5 respectively, as being shown in Figure 4 (a) ∼ (c). The absorption peak further red shifts to 461 nm as well as 558 nm for the composition of x = 0.75 and x = 1 respectively, as being shown in Figure 4 (d) ∼ (e). The band gap energies of CdSe_1–x_S_x_ nanocrystals red shift to longer wavelength side when the concentration of Se sources increased, driving the absorption peak approaching to that of pure CdSe nanocrystals. In other words, the more S source is contained in the CdSe_1–x_S_x_ nanocrystals, the larger is its band gap energy and the shorter wavelength is the absorption peak for the CdSe_1–x_S_x_ nanocrystals. Therefore chemical composition provides us an additional degree to tune the band gap energies of ternary semiconductor nanocrystals except the traditional quantum size effect. Of course this paper is not the first one to tune the band gap energies of semiconductor nanocrystals by chemical composition, but it is the first one for fabrication of water soluble CdSe_1–x_S_x_ nanocrystals. It is a facile green and environment friendly way to synthesize CdSe_1–x_S_x_ nanocrystals with very low cost.

### 1.4. Photoluminescence spectra

**Figure 5:**
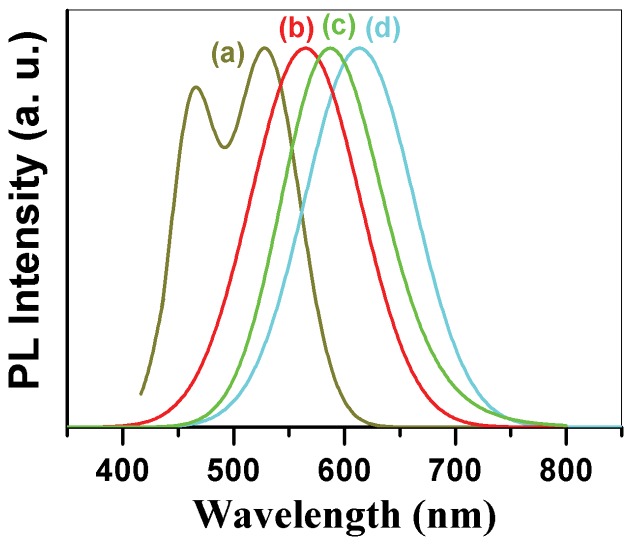
Tunable photoluminescence spectra of CdSeS nanocrystals with particle size. CdSeS nano-specimens were heat treated at (a) 80°C, (b) 180°C, (c) 220°C, (d) 260°C, all for 5 minutes.

Figure 5 shows the photoluminescence spectra of as-prepared *CdSe_0.2_S_0.8_* nanocrystals which were heat treated at different temperatures. The coexistence of CdS nanocrystals and CdSe nanocrystals at 80°C, represented by a narrow PL signal at 464 nm and a broad PL signal at 528 nm, respectively, was observed in Figure 5a (*80°C)*. The narrow PL signal at 464 nm was correlated to the emission peak of the CdS nanocrystals by PLE measurements and thus proving the origin of the blue luminescence, while the broaden signal at 528 nm was corresponding to the emission spectrum of CdSe nanocrystals (Figure 5a). This result indicates that single phase *CdSe_0.2_S_0.8_* was not formed at the beginning of the reaction (80*°C)*, instead a mixture of CdS and CdSe were the initial products. The other possibility is the coexistence of smaller and larger particles with two size distribution centers.

This possibility for the appearance of two emission peaks in Figure 5 (a) is the equilibrium between smaller and larger particles being related to the two peaks at 464 nm and 528 nm respectively. The small *CdSe_0.2_S_0.8_* nanoparticles act as growth material or even as nuclei for the larger NCs after raising the temperature from 80°C to 260°C. Transmission electron microscopy (TEM) observation of the above-mentioned equilibrium confirmed the existence of spherical particles with two mean diameters of 3.2 nm and 5 nm (Figure 2 (c) & (d)), which is consistent with the values calculated from the spectroscopic data according to Yu et al. [42]. The larger particles exhibited a broad size distribution, whereas the smaller particles showed an unexpected narrow size distribution. This can be concluded from the FWHM of the corresponding PL spectra.

The clear evidence that small cubic *CdSe_0.2_S_0.8_* nanocrystals differ significantly from conventional NCs can be seen by raising the reaction temperature from 80°C to 260°C (Figure 4). Subsequently, the narrow peak of the small cubic NCs decreases without showing any red shift while the broad peak shifts to longer wavelength side, indicating that the growth of NCs was at the cost of smaller particles. The transition from two emission peaks to one peak accompanying with the disappearance of the first small emission peak in the photoluminescence spectra was observed in Figure 5 (a) and (b) when the heat treatment temperature of the specimen rises from 80°C to 180°C. This transition may also implicate the formation of single phase CdSe_0.2_S_0.8_ nanocrystals, which should be able to trig only one emission peak. The broad peak at 528 nm red shifts to 563 nm when the temperature of the heat treatment for the suspension solution of *CdSe_0.2_S_0.8_* nanocrystals was raised from 80°C to 180°C (Figure 5 b), which further shifts to 586 nm after the heat treatment of the specimen at higher temperature (220°C) for 5 minute (Figure 5c). Finally the PL emission peak red shifts to 614.5 nm when the specimen was heat treated at 260°C for 5min, as being shown in Figure 5 (d). This single PL emission peak should reflect the electron transition from the valence band to conduction band in single phase *CdSe_0.2_S_0.8_* nanocrystals instead of mixture of CdSe and CdS nanocrystals. This result is in good agreement with the formula of *CdSe_0.2_S_0.8_* compound being drawn from XRD pattern. Similar results have been reported by Kasuya et al. [43], Chen [44] and Riehle et al. [37] who studied the transformation of magic size CdSe clusters into CdSe NCs by UV-vis absorption spectroscopy and photoluminescence spectra. Again the big resolved quantum confinement based on photoluminescence spectra is due to the larger particle size of cubic *CdSe_0.2_S_0.8_* nanocrystals instead of ultrasmall particle size for pure CdSe nanocrystals. This point differs significantly from the results being observed by Riehle et al. [37], who assigned the strong quantum confine effect in PL spectra to ultra small particle size of pure CdSe nanocrystals, which were also fabricated using MPA as the surface activation agent. Actually the S ions inside MPA molecule may release additional S sources, which would combine with Se source to react with Cd precursor together, thus CdSeS instead of pure CdSe nanocrystals could be formed.

The PL quantum yield efficiency of CdSeS nanocrystals was found to develop with a time constant of 5 days from zero to terminal saturation value of approximate 49%, which was maintained constant within the remaining time of several months.

## Materials and Methods

First of all, a stock solution of Se-S precursor was prepared by dissolving 0.0154 g Na_2_SeO_3_ powder in 2 mL distilled water, afterwards 20 mL glycerin was put into the above solution. Then 1.0078 g glucose powder dissolving in 2 mL distilled water together with 1mL 3-mercaptopropionic acid (MPA) was added into above solution at 80°C. Glucose was used as reduction agent to produce Se source, while MPA plays both roles of surface activating agent and providing the source of S. The mixture solution was heated up to 260°C for 20 minites, the color of the solution changes from limpidity, yellow, orange, red to dark red at different temperatures of heat treatment. The colorful suspension comes from the formation of Se nanoparticles, which was reduced from Na_2_SeO_3_ by glucose. Afterwards the red suspension solution was cooling down to room temperature. In order to fabricate homogeneous CdSeS nanocrystals, a transparent Se precursor is necessary. Therefore the red suspension solution was heated up to 220°C again, the Se nanoparticles were dissolved in the solution. However, after the precursor solution was cooling down to room temperature, a few black suspended colloids could be observed in the limpidity solution. In this case, 10 mL more distilled water was added into the suspension solution, which was heated up to 260°C once more. At this point, the black suspended colloids disappeared and the suspension becomes colorless transparent solution after cooling down to room temperature. The clear solution was stored as Se-S precursor.

0.0211 g CdCl_2_*2.5H_2_O powder and 0.2166 g polyethylene glycol were dissolved together in 15 mL distilled water in a three neck flask at 60°C, afterwards 0.1 mL MPA and 10 mL 1 M NaOH were added into the above solution, which was used as the stock solution of Cd precursor. The Cd stock solution was maintained at 80°C, into which the stock solution of Se-S precursor was injected slowly. Immediately CdSeS nanocrystals were formed, the solution turned into transparent yellow one. 5 mL of the specimen was taken out and put in a small glass bottle for optical and microstructure measurement. The remaining suspension keeps heating at 120°C, 180°C and 260°C, respectively. At each temperature point, 5 mL colloid suspension solution was taken out from the flask for optical measurement. There was no evaporation but some refluxing in the reaction solution. The above process was carried out under ambient atmosphere. The colloid dispersion solution exhibits excellent stability, nanoparticles didn't deposit onto the bottom of the bottle or the color of the solution didn't change for several months. The glycerin and MPA formed a macromolecule frame network, on which nanocrystals were pinned up making the dispersion solution of CdSeS nanocrystals extremely stable. The as-prepared products were characterized by using various methods. The sample for the X-ray diffraction (XRD) was prepared by centrifugation of the solution with distilled water at 12000 rpm for 30 min. The structural characterization of the CdSeS nanocrystals was monitored by Rigaku powder X-ray diffraction machine. The microstructure feature of the CdSeS nanocrystals was performed upon a JEOL 2100F high resolution transmission electronic microscopy (HRTEM). UV–vis absorption spectra for the suspension solution of CdSeS nanocrystals has been recorded by a Phoenix 1900PC UV-Vis-NIR Spectrophotometer and fluorescence spectrum by USB 4000 spectroscopy made by Ocean optics.

## Conclusion

In summary, we have demonstrated a phosphine-free protocol for synthesizing water soluble CdSeS nanocrystals. It employed glycerin to facilitate Cd (CH_3_COO)_2_ powder dissolution in water at room temperature; phosphine-free Na_2_SeO_3_ precursor in polyethylene glycol and MPA as Se-S sources have been successfully applied toward the synthesis of CdSeS nanocrystals in zinc blende structure. Glucose was used as reduction agent to produce Se source, XRD pattern and HRTEM images confirmed the formation of cubic CdSe_0.2_S_0.8_ nanocrystals, whose UV-Vis absorption peaks locate within the range of 335 nm∼432 nm. This shouldn't reflect ultra-small particle size of pure CdSe nanocrystals, but actually is corresponding to the quantum confinement effect of cubic CdSe_0.2_S_0.8_ nanocrystals. Tunable band gap energies of series water soluble CdSe_1–x_S_x_ have been achieved through chemical composition upon atomic ratio of Se/S sources, which come from the Na_2_SeO_3_ and Na_2_S solution. The corresponding PL spectra revealed band-edge luminescence for CdSe_0.2_S_0.8_ QDs of all size range, whose PLE peaks cover from 464 nm to 614.5 nm; no deep trap luminescence was detected.
